# Spotlight on *FAM72B*: Pan-Cancer Expression Profiles and Its Potential as a Prognostic and Immunotherapeutic Biomarker

**DOI:** 10.3390/genes16101140

**Published:** 2025-09-26

**Authors:** Anran Chu, Yuchan Wang

**Affiliations:** Department of Pathogenic Biology, School of Medicine, Nantong University, No.19 Qixiu Road, Nantong 226001, China

**Keywords:** *FAM72B*, pan-cancer, tumor immune microenvironment, prognosis, biomarker, immunotherapy

## Abstract

**Background/Objectives:** *FAM72B* (Family with sequence similarity 72 member B) is a gene whose function is not yet fully elucidated and which belongs to the *FAM72* gene family. Recent studies have indicated that it is involved in the regulation of stem cell proliferation and DNA repair and serves as a valuable prognostic biomarker for a few types of cancer. This study aimed to systematically investigate the expression profile of *FAM72B* in pan-cancer, its role in the tumor immune microenvironment, and its potential as a prognostic and immunotherapeutic biomarker. **Methods:** Using bioinformatics tools such as SangerBox3.0, GEPIA2.0, Kaplan–Meier Plotter, and cBioPortal, we systematically analyzed the correlation of *FAM72B* expression levels with various cancer types, clinical pathological parameters, prognostic value, genetic mutations, genomic heterogeneity, immune checkpoint genes, immune cell infiltration levels, and single-cell-level characteristics. **Results:** *FAM72B* was found to be overexpressed in most cancers and significantly associated with poor prognosis, although it may exert a protective effect in some cancers like thymoma (THYM). Its expression level was positively correlated with tumor mutation burden (TMB), microsatellite instability (MSI), neoantigen (NEO) levels, and expression of immune checkpoint genes in most cancers, suggesting that patients with high *FAM72B* expression may respond better to immune checkpoint inhibitors. Moreover, *FAM72B* expression was significantly correlated with the infiltration levels of various immune cells in the tumor immune microenvironment across pan-cancer. Single-cell sequencing results also demonstrated a significant correlation between *FAM72B* and the biological functional states of multiple cancers. **Conclusions:** *FAM72B* holds promise as a potential pan-cancer prognostic biomarker and therapeutic target, providing a novel basis for the development of personalized treatment strategies.

## 1. Introduction

Family with sequence similarity 72 (*FAM72*) includes four members: *FAM72A*, *FAM72B*, *FAM72C*, and *FAM72D*. *FAM72A* (also known as *Ugene*, *LMPIP*, or *p17*) is a highly conserved gene in multicellular organisms, located on human chromosome 1q32.1 [1,2,3,4]. It plays crucial roles in cell cycle regulation, DNA repair and antibody maturation, neural stem cell regulation, and tumorigenesis. *FAM72A* expression is regulated by FoxM1 and APC/C at both transcriptional and post-transcriptional levels, and the gene can bind to tubulin and PP2A-B56 subunits to modulate tubulin and Mcl1 phosphorylation, thereby affecting cell cycle progression and apoptosis signaling [5]. In antibody maturation, *FAM72A* antagonizes uracil DNA glycosylase 2 (UNG2) to promote error-prone DNA repair, which is vital for B cell class-switch recombination and somatic hypermutation [6,7,8,9]. In neural stem cells, *FAM72A* cooperates with the Srgap2 gene to regulate cell differentiation and proliferation [3,4,10]. Additionally, *FAM72A* is overexpressed in various cancers, such as glioma (including glioblastoma multiform) [11,12], lung adenocarcinoma (LUAD) [13], hepatocellular carcinoma (HCC) [14,15], breast invasive carcinoma (BRCA) [16], and uterine corpus endometrial carcinoma (UCEC) [17], and is associated with poor prognosis, serving as a prognostic biomarker for multiple cancers. Pan-cancer analyses have been conducted on it [18]. The *FAM72C* gene has been found to be located on chromosome 1q21.2 [4]. Presently, there is limited understanding of the function of the protein encoded by *FAM72C*, as well as its specific roles and molecular mechanisms in cell cycle regulation and DNA repair processes. In disease-associated studies, *FAM72C* has been identified as a potential therapeutic target in systemic lupus erythematosus (SLE) through Bayesian gene network analysis [19]. Additionally, the DNA methylation status of *FAM72C* has been detected as a candidate molecular marker for blood-based detection in colorectal cancer (CRC) [20,21]. The results of uni- and multivariate Cox regression analyses, along with the expression levels in oral squamous cell carcinoma (OSCC) tissues and cells, indicate that *FAM72C*, like *FAM72B*, is considered a promising molecular marker for the prognosis and therapeutic targeting of OSCC patients [22]. *FAM72D*, located in the 1q21.1 region of the human chromosome, has demonstrated significant potential as a prognostic biomarker in multiple myeloma [23], prostate cancer [24], kidney renal clear cell carcinoma (KIRC) [25], HCC [26], and LUAD [27]. In multiple myeloma, the DNA demethylation of the *FAM72D* gene is closely associated with enhanced tumor cell proliferation and can serve as a prognostic biomarker for this malignancy. *FAM72D* functions as part of the FOXM1 transcription factor network, and its high expression along with FOXM1 renders cells more susceptible to epigenetic drugs targeting histone deacetylases and DNA methyltransferases [23]. In the study of HCC, exosome-derived *lnc-FAM72D-3* [26], which serves as a diagnostic and prognostic biomarker, promotes cytoskeleton remodeling via the MBNL1/FAK axis, thereby enhancing HCC cell resistance to lenvatinib and elucidating the molecular mechanisms underlying HCC drug resistance [28]. Compared to the other three members of the *FAM72* family, the intronic variations of FAM72B, coupled with its unique evolutionary history [4], endow it with potential significant functions in cancer research and pave the way for novel perspectives and innovative research directions in pan-cancer analysis. Previous studies have demonstrated that *FAM72B*, located at 1p11.2 of the chromosome, can function as a diagnostic and prognostic biomarker for various solid tumors, including LUAD [29], OSCC [22], and prostate adenocarcinoma (PRAD) [30,31]. Additionally, subsets of triple-negative breast cancer (TNBC) cells expressing *FAM72B* have been preliminarily identified as being resistant to paclitaxel, suggesting that *FAM72B* may play a crucial role in tumor resistance mechanisms [32,33]. Recent studies have further indicated that this gene is also involved in the regulation of stem cell proliferation and DNA repair [33,34]. Precisely, similar to *FAM72A*, *FAM72B* exhibits similar molecular interactions, such as binding to UNG and RPA proteins, suggesting its involvement in DNA repair and replication stress response, akin to *FAM72A*’s role in inhibiting base excision repair (BER) during class-switch recombination (CSR). However, *FAM72B*’s increased chromatin binding during the S phase and following DNA damage, as well as its regulatory role in RPA2 phosphorylation after replication stress, reveal potential unique functional mechanisms. Additionally, functional validation experiments show that in *FAM72B*-depleted cells, phosphorylation of RPA1 on serine residues 4 and 8 is increased, further confirming *FAM72B*’s crucial role in avoiding or dealing with replication stress. These findings provide new insights into cancer research, particularly in DNA repair and tumor resistance mechanisms [34]. However, the prognostic value and potential functions of *FAM72B* in the pan-cancer immune microenvironment remain to be elucidated.

Although the mortality rate of cancer has shown a declining trend in recent years, the incidence of new cases has significantly increased, indicating the ongoing challenges in cancer control and prevention [35,36].

Since its inception, pan-cancer analysis has attracted widespread attention [37,38] and has recently achieved a milestone in elucidating the molecular commonalities across different cancer types [39]. However, pan-cancer analysis still faces numerous challenges, such as enhancing the precision of pan-cancer treatments and deciphering the complex interactions within the tumor microenvironment. Future research needs to further explore these issues, develop new technologies and methods, and promote the in-depth development of pan-cancer studies.

The tumor immune microenvironment (TIME) primarily involves interactions between tumor cells and immune cells; within this ecological niche, immune cells and immune-related molecules exert crucial influences [40]. The expression levels of immune checkpoint molecules can modulate the activity of immune cells, thereby influencing the immune response to tumors [41]. The spatial distribution of infiltrating immune cells within the TIME determines the prognosis of cancer patients [42]. Immune checkpoint blockade therapy has revolutionized the landscape of cancer treatment [43]. The infiltration and functional status of immune cells in tumor tissues are key factors for immunotherapy [44,45,46]. A comprehensive exploration of the spatiotemporal heterogeneity of the TIME will provide possibilities for the development of personalized cancer immunotherapies [46,47,48].

This study analyzed the expression patterns and clinical significance of *FAM72B* in cancers using bioinformatics techniques, thereby providing further evidence to help us better understand the importance of *FAM72B* in the tumor immune microenvironment across various types of cancer.

## 2. Materials and Methods

### 2.1. Data Collection and Processing

Using the SangerBox3.0 platform (http://sangerbox.com/), we retrieved a pan-cancer dataset from the UCSC database that had undergone uniform standardization and excluded cancer types with fewer than three samples; subsequently extracted and filtered the expression data of the *FAM72B* gene across these samples; applied a log2(x + 1) transformation to the expression values; calculated the expression differences of FAM72B under different conditions using relevant R software packages; conducted significance difference analyses for clinicopathological parameters and prognostic survival indicators using the unpaired Wilcoxon rank-sum test and the Log-rank test, respectively; and assessed the correlations between the expression of *FAM72B* and the levels of TMB, MSI, NEO, immune checkpoint expression, and immune cell infiltration using the Pearson correlation coefficient. Among these analyses, the pan-cancer dataset used for clinicopathological parameters and genomic heterogeneity analyses was sourced from the TCGA database (PANCAN, N = 10,535, G = 60,499), while the datasets for other analyses were sourced from the TCGA, TARGET, and GTEx databases (PANCAN, N = 19,131, G = 60,499).

### 2.2. mRNA Expression of FAM72B in Pan-Cancer

Visualization maps and bar charts of the consensus dataset, which was integrated by normalizing and combining data from the HPA and GTEx datasets, involving 20,162 genes and encompassing RNA expression data from 50 human tissues, were retrieved from the Human Protein Atlas (HPA) 24.0 database (https://www.proteinatlas.org/) to illustrate the expression levels of *FAM72B* in normal human tissues. On the SangerBox3.0 platform, the expression data of the *FAM72B* gene (ENSG00000188610) from the pan-cancer dataset (PANCAN, N = 19,131, G = 60,499) were extracted for each sample. Subsequently, samples originating from Solid Tissue Normal, Primary Solid Tumor, Primary Tumor, Normal Tissue, Primary-Blood-Derived Cancer—Bone Marrow, and Primary-Blood-Derived Cancer—Peripheral Blood were selected, and their expression values were subjected to log2(x + 1) transformation. The expression differences of FAM72B between normal and tumor samples, as well as across samples from various clinicopathological parameters (including gender, TNM stage, disease stage, and grade), were calculated for each type of cancer using R software (version 3.6.4). The expression differences of FAM72B between the normal and tumor groups were calculated using the non-parametric Wilcoxon rank sum and signed rank tests, with a significance threshold of *p* < 0.05, to determine statistical significance. When targeting clinicopathological parameters, Primary-Blood-Derived Cancer—Peripheral Blood and Primary Tumor were the final sample sources that were selected. Additionally, the Kruskal–Wallis test (kruskal.test) was employed to assess expression differences across multiple groups of samples.

### 2.3. Pan-Cancer Survival Analysis of FAM72B

On the SangerBox3.0 platform, the forest plot obtained from the Cox proportional hazards regression model established using the coxph function from R software (version 3.2-7) was used to evaluate the associations between *FAM72B* expression and overall survival (OS), disease-specific survival (DSS), disease-free interval (DFI), and progression-free interval (PFI) in pan-cancer. The analysis was performed in a univariate manner, focusing solely on the impact of gene expression on prognosis without considering other confounding variables, but this approach may limit the robustness of the results and thus has certain limitations. Future studies should consider employing multivariate Cox regression analysis to enhance the robustness of the findings. The Log-rank test was employed for statistical analysis to assess the significance of prognosis. Before plotting, cancer types with fewer than 10 samples were excluded. The supplementary prognostic relationships for *FAM72B* were visualized using the Kaplan–Meier Plotter (https://kmplot.com/analysis/) and GEPIA2.0 (http://gepia2.cancer-pku.cn/#index) databases. The median value was selected as the group cutoff to split high- and low-expression cohorts of *FAM72B*, with a significance threshold of *p* < 0.05 to determine statistical significance. The significance of expression differences in *FAM72B* was assessed using the hazard ratio (HR) and its corresponding 95% confidence interval (95% CI).

### 2.4. Analysis of FAM72B Genetic Mutations

To obtain information on *FAM72B* gene mutation types, sites, frequencies, and survival differences, the “Cancer Types Summary”, “Mutations”, and “Comparison/Survival” modules were selected from the cBioPortal6.3.6 database (https://www.cbioportal.org/). The data were derived from 32 cancer types within the PanCancer Atlas datasets of the TCGA database, involving 10,967 patients. cBioPortal integrates and standardizes variant interpretation data from multiple external databases, including Genome Nexus, CIViC, and COSMIC [49]. It categorizes mutation types based on the variant classification field in Mutation Annotation Format (MAF) files [49]. To enhance the biological relevance of analyses and minimize noise, cBioPortal filters out mutations with less biological significance by default, such as silent and intronic mutations. Additionally, cBioPortal employs tools like Mutation Assessor to predict and evaluate the functional impact of mutations [50]. In survival analysis, cBioPortal utilizes standardized next-generation sequencing (NGS) data and employs bioinformatics tools such as MuTect and VarScan for mutation detection. Samples in which mutations are detected are categorized into the “altered group”, whereas those without detected mutations are assigned to the “unaltered group”. Subsequently, the Log-rank test is utilized to compare survival differences between the two groups, with a significance threshold of *p* < 0.05 set to assess the significance of these differences.

### 2.5. Tumor Mutational Burden, Microsatellite Instability, and Neoantigen

MSI and NEO scores for each type of cancer were obtained from previous studies [51,52], while TMB scores were calculated using the tmb function from the R package maftools (version 2.8.05), and all three indicators were visualized using lollipop plots.

### 2.6. Analysis of Immune Checkpoint Genes and Immune Cell Infiltration in the Tumor Immune Microenvironment

Immune checkpoint gene data were obtained from previous studies [52]. Infiltration scores for various immune cell subtypes in each patient within each tumor type were calculated using the CIBERSOR algorithm from the R package IOBR (version 0.99.9), and for each independent tumor type, Pearson’s correlation coefficients between *FAM72B* gene expression levels and infiltration scores of all immune cell subtypes were subsequently computed using the corr. test function from the R package psych (version 2.1.6) to identify significantly correlated immune infiltration scores.

### 2.7. FAM72B-Related Gene Enrichment Analysis

We identified 30 genes related to *FAM72B* via the STRING12.0 database (https://cn.string-db.org/), constructed a protein–protein interaction (PPI) network for these genes using Cytoscape3.9.1 (https://cytoscape.org/) with a confidence score threshold of R > 0.4, and subsequently performed Gene Ontology (GO) enrichment analysis on these genes using the R packages clusterProfiler and pathview.

### 2.8. Single-Cell Analysis of FAM72B Gene Expression

We explored the functional states of *FAM72B* across multiple cancer types using the CancerSEA database (http://biocc.hrbmu.edu.cn/CancerSEA/) and visualized its single-cell expression patterns in pan-cancer analysis through t-distributed Stochastic Neighbor Embedding (T-SNE) plots generated from the database data.

## 3. Results

### 3.1. Analysis of FAM72B mRNA Expression and Its Correlation with Clinicopathological Parameters in Multiple Human Cancers

**Figure 1:**
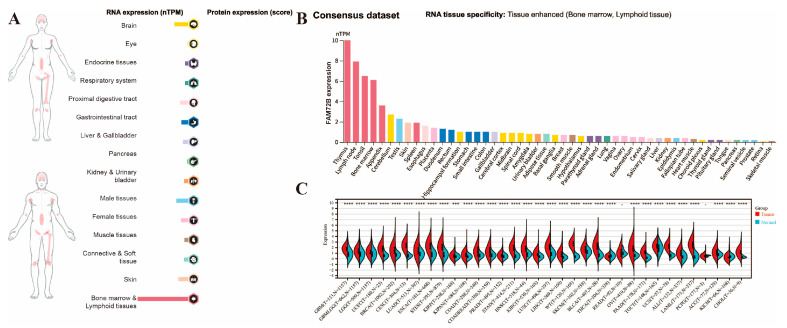
mRNA expressions of the *FAM72B* gene in normal human tissues and various cancers. (**A**) An overview of *FAM72B* mRNA and protein expression profiles across human organs and tissues. (**B**) Summaries of *FAM72B* mRNA expression in diverse human organs and tissues, derived from the consensus dataset. (**C**) *FAM72B* expression profiles in 34 cancerous and corresponding adjacent tissues from the TCGA, TARGET, and GTEx databases. Expression differences of FAM72B between normal and tumor groups were analyzed using non-parametric Wilcoxon rank sum and signed rank tests (*** *p* < 0.001, **** *p* < 0.0001).

Initially, we analyzed the mRNA expression of *FAM72B* in 50 types of normal human tissues using the Human Protein Atlas (HPA) database (Figure 1A,B). Analysis of the visual atlas (Figure 1A) and consensus dataset (Figure 1B) revealed that, although the overall expression level of *FAM72B* in normal tissues was low, it was highest in bone marrow and lymphoid tissues (specifically the thymus, lymph node, tonsil, bone marrow, and appendix), where it exhibited tissue-specific expression. Conversely, *FAM72B* expression was lowest in the retina and skeletal muscle. Because of the small number of normal tissues in the TCGA database, we combined this database and the GTEx database using the pan-cancer platform SangerBox3.0 to analyze the *FAM72B* expression in 34 types of human cancers. The results demonstrated that the mRNA expression levels of *FAM72B* were significantly upregulated in 30 types of cancer compared to their corresponding paraneoplastic tissues, while they were downregulated in THCA and KICH. However, no significant differences were observed in READ and PCPG (Figure 1C, Table 1).

**Figure 2:**
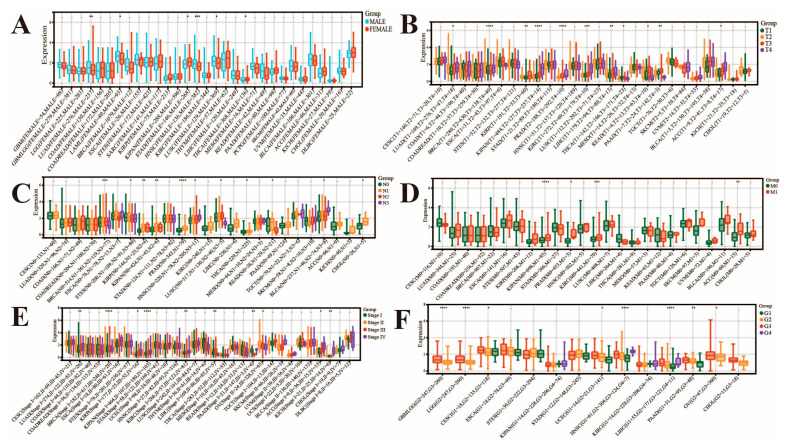
Correlations of *FAM72B* mRNA expression levels with clinical characteristics. Correlations between *FAM72B* and gender (**A**), T category (**B**), N category (**C**), M category (**D**), stage (**E**), and grade (**F**). The statistical significance of pairwise differences was assessed using the non-parametric Wilcoxon rank sum and signed rank tests, and expression differences across multiple groups were determined using the Kruskal–Wallis test (kruskal.test) (* *p* < 0.05, ** *p* < 0.01, *** *p* < 0.001, **** *p* < 0.0001).

Then, we evaluated the correlation between *FAM72B* expression and clinicopathological parameters, including gender, TNM stage, disease stage, and histological grade, in multiple human cancers using the pan-cancer platform SangerBox3.0 (Figure 2). We found that this expression was significantly higher in female than male patients in STAD and THCA, while the opposite was true in HNSC, LAML, LUAD, and LUSC (Figure 2A). In the context of TNM staging, significant differences were observed across 16 types of cancers, including BRCA, KIPAN, PRAD, KIRC, KIRP, LIHC, PAAD, ACC, READ, THCA, LUAD, STES, HNSC, LUSC, BLCA, and CHOL (Figure 2B–D). In detail, as for the T category, *FAM72B* exhibited higher expression in highly aggressive states of KIPAN, KIRC, PRAD, BRCA, ACC, LUAD, and LIHC; moreover, there was a trend of increasing *FAM72B* expression with progressive tumor staging in ACC, LUAD, and LIHC. Conversely, *FAM72B* showed higher expression in the less aggressive states of THCA and READ (Figure 2B). In terms of the N category, *FAM72B* expression was highest in BLCA and HNSC, where the cancer had metastasized to a greater number of more distant lymph nodes. Additionally, the box plots indicated that *FAM72B* expression was highest in BRCA and LUSC in the N2 stage, whereas that in PRAD, KIPAN, KIRP, THCA, READ, STES, and CHOL was highest in the N1 stage (Figure 2C). With regard to the M category, we observed significant differences in four types of tumors, including KIPAN, KIRC, ACC, and PRAD, and moreover, M1 exhibited elevated expression levels of *FAM72B* compared to M0 (Figure 2D). The above results suggested that patients, who were in advanced stages of KIPAN, KIRC, ACC, and PRAD cancers, tended to exhibit higher levels of *FAM72B* expression, which typically indicated that this gene was associated with tumor progression and may have been indicative of a poorer prognosis. In addition, elevated *FAM72B* expression is also observed in late-intermediate-stage BRCA, KIRP, HNSC, LUAD, LIHC, LUSC, and BLCA, as well as in intermediate-stage THCA and READ. Moreover, in clinical staging, higher *FAM72B* expression is observed in patients with advanced-stage KIPAN, KIRC, KIRP, LUAD, and ACC, and in particular, there is a rising trend in ACC staging, implying a potential relation to the disease progression of ACC. Additionally, we observed peak *FAM72B* expression in patients with stage II BRCA and OV and stage III LIHC, with a decline in expression accompanying the progression of OV stage (Figure 2E). Furthermore, it is noted that *FAM72B* expression is markedly upregulated in high-grade (III–IV) compared to low-grade (I–II) tumors in HNSC, LIHC, GBM, GBMLGG, PAAD, UCEC, CESC, and OV. Notably, the upward trend in expression accompanying the increased grading of HNSC, UCEC, PAAD, GBM, GBMLGG, and OV implies a potential role for *FAM72B* in the disease progression of these cancers (Figure 2F).

### 3.2. Impact of FAM72B mRNA Expression on Prognosis in Multiple Human Cancers

**Figure 3:**
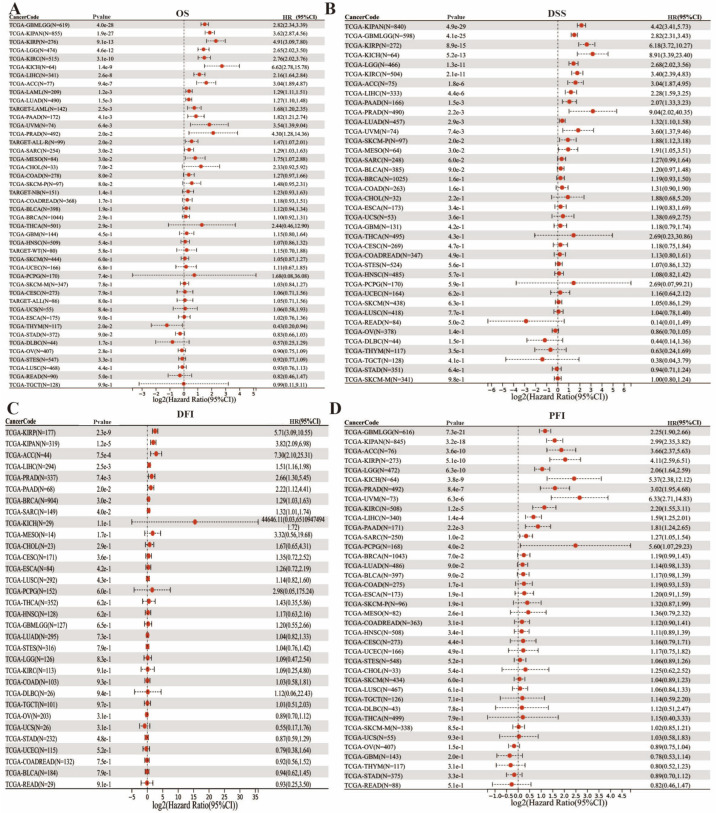
Relationships between *FAM72B* expression and overall survival (OS) (**A**), disease-specific survival (DSS) (**B**), disease-free interval (DFI) (**C**), and progression-free interval (PFI) (**D**) in human pan-cancer via the pan-cancer platform SangerBox3.0. The correlation between *FAM72B* expression and prognostic indicators was evaluated using univariate Cox regression analysis, and prognosis significance was evaluated using the Log-rank test for statistical analysis.

Subsequently, we conducted an analysis to determine the correlation between the expression levels of *FAM72B* mRNA and the prognostic outcomes in oncological patients. The initial findings from the pan-cancer platform SangerBox3.0 revealed that, in the OS analysis, elevated *FAM72B* expression was associated with a favorable prognosis in THYM, whereas this association was reversed in 16 other cancer types, including GBMLGG, LGG, KIRP, KIPAN, KIRC, LIHC, ACC, KICH, LAML, LUAD, uveal melanoma (UVM), PAAD, sarcoma (SARC), PRAD, mesothelioma (MESO), and ALL (from the TARGET database) (Figure 3A). The DSS analysis data indicated that high expression of *FAM72B* was significantly correlated with an adverse prognosis in 14 distinct tumor types, including GBMLGG, LGG, KIRP, KIPAN, KIRC, LIHC, ACC, KICH, LUAD, PRAD, UVM, PAAD, MESO, and skin cutaneous melanoma-primary (SKCM-P) (Figure 3B). The DFI results showed that overexpression of *FAM72B* was related to poor prognosis in KIRP, KIPAN, ACC, PRAD, LIHC, BRCA, SARC, and PAAD (Figure 3C), while the PFI results demonstrated that its increased expression was correlated with an unfavorable prognosis in GBMLGG, LGG, KIRP, KIPAN, PRAD, KIRC, UVM, ACC, KICH, LIHC, PAAD, SARC, and PCPG (Figure 3D). Meanwhile, utilizing the GEPIA2.0 database with the median *FAM72B* expression value as the threshold, our analysis revealed that OS and recurrence-free survival (RFS) were both decreased in cancers with elevated *FAM72B* expression in ACC, KIRP, LGG, LIHC, and SARC, suggesting a correlation between high *FAM72B* expression and adverse prognosis in these cancers (Figure S1A–J). In addition, poor prognosis in terms of OS for KIRC, LUAD, and MESO, as well as RFS for KICH, PAAD, PRAD, and UVM, is also associated with a high expression of *FAM72B* (Figure S1K–Q). Interestingly, elevated *FAM72B* expression was potentially linked to improved prognosis in LUSC (OS: HR = 0.7, *p* = 9.1e−3) and THYM (OS: HR = 0.21, *p* = 3.9e−2) (Figure S1R,S).

**Figure 4:**
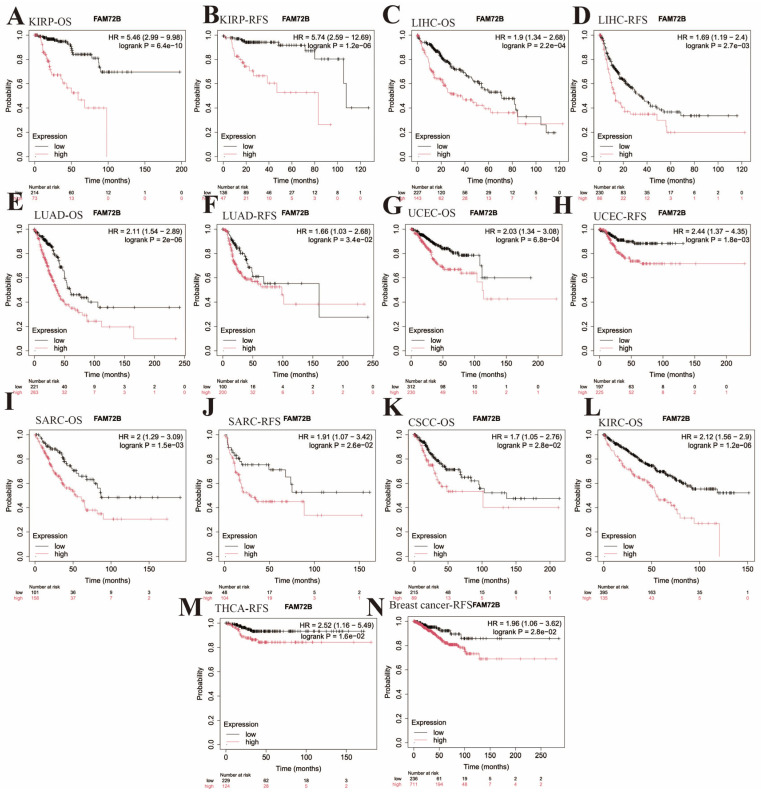
Kaplan–Meier survival curve analysis of the poor prognostic significance of the high expression of *FAM72B* in 9 types of human cancers using the Kaplan–Meier plotter database. (**A**–**N**) The correlation of high *FAM72B* expression with poor OS and RFS in KIRP (**A**,**B**), LIHC (**C**,**D**), LUAD (**E**,**F**), UCEC (**G**,**H**), and SARC (**I**,**J**); with just poor OS in CSCC (**K**) and KIRC (**L**); and with just poor RFS in THCA (**M**) and breast cancer (**N**). The median value was selected as the group cutoff to split high- and low-expression cohorts of *FAM72B*; a significance threshold of *p* < 0.05 was chosen to determine statistical significance. The significance of expression differences in *FAM72B* was assessed using HR and its corresponding 95% CI. OS—overall survival; RFS—recurrence-free survival; KIRP—kidney renal papillary cell carcinoma; LIHC—liver hepatocellular carcinoma; LUAD—lung adenocarcinoma; UCEC—uterine corpus endometrial carcinoma; SARC—sarcoma; CSCC—cervical squamous cell carcinoma; KIRC—kidney renal clear cell carcinoma; THCA—thyroid carcinoma; HR—hazard ratio; CI—confidence interval.

**Figure 5:**
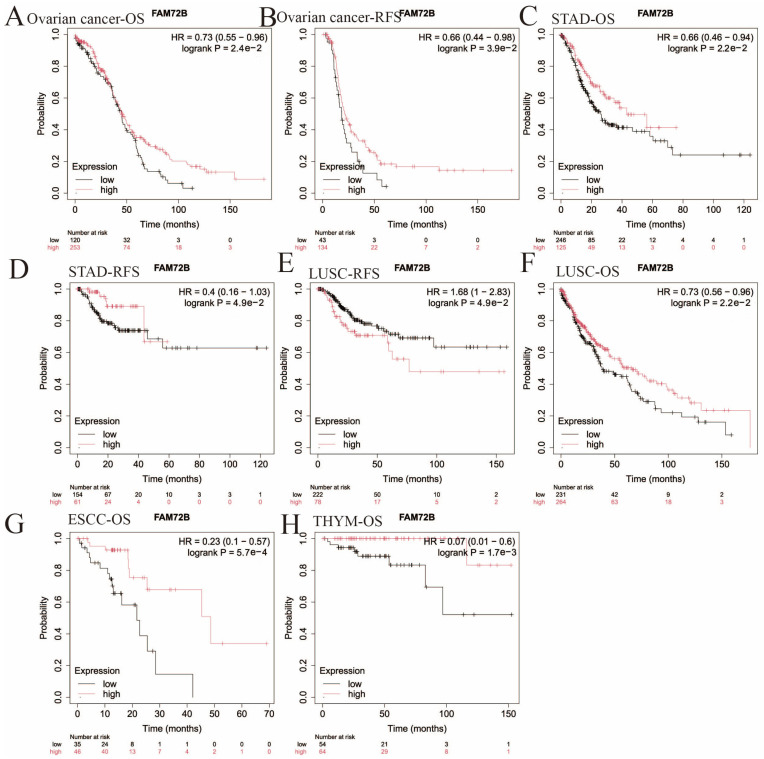
Kaplan–Meier survival curve analysis of the better prognostic significance of the high expression of *FAM72B* in 5 types of human cancers using the Kaplan–Meier plotter database. (**A**–**H**) Correlations of high *FAM72B* expression with better OS and RFS in ovarian cancer (**A**,**B**) and STAD (**C**,**D**); with poor RFS (**E**) and better OS (**F**) in LUSC; and with better OS in ESCC (**G**) and THYM (**H**). The median value was selected as the group cutoff to split high- and low-expression cohorts of *FAM72B*; a significance threshold of *p* < 0.05 was used to determine statistical significance. The significance of expression differences in *FAM72B* was assessed using HR and its corresponding 95% CI. OS—overall survival; RFS—recurrence-free survival; STAD—stomach adenocarcinoma; LUSC—lung squamous cell carcinoma; ESCC—esophageal squamous cell carcinoma; THYM—thymoma; HR—hazard ratio; CI—confidence interval.

Furthermore, we employed the Kaplan–Meier plotter database to assess the prognostic value of *FAM72B* expression in 21 types of human tumors (Figure 4 and Figure 5, Table 2). Elevated *FAM72B* expression was associated with a more unfavorable prognosis in KIRP (OS: HR = 5.46, 95% CI = 2.99 to 9.98, *p* = 6.4e−10; RFS: HR = 5.74, 95% CI = 2.59 to 12.69, *p* = 1.2e−6), LIHC (OS: HR = 1.9, 95% CI = 1.34 to 2.68, *p* = 2.2e−4; RFS: HR = 1.69, 95% CI = 1.19 to 2.4, *p* = 2.7e−3), LUAD (OS: HR = 2.11, 95% CI = 1.54 to 2.89, *p* = 2e−6; RFS: HR = 1.66, 95% CI = 1.03 to 2.68, *p* = 3.4e−2), SARC (OS: HR = 2, 95% CI = 1.29 to 3.09, *p* = 1.5e−3; RFS: HR = 1.91, 95% CI = 1.07 to 3.42, *p* = 2.6e−2), UCEC (OS: HR = 2.03, 95% CI = 1.34 to 3.08, *p* = 6.8e−4; RFS: HR = 2.44, 95% CI = 1.37 to 4.35, *p* = 1.8e−3), breast cancer (RFS: HR = 1.96, 95% CI = 1.06 to 3.62, *p* = 2.8e−2), THCA (RFS: HR = 2.52, 95% CI = 1.16 to 5.49, *p* = 1.6e−2), cervical squamous cell carcinoma (CSCC) (OS: HR = 1.7, 95% CI = 1.05 to 2.76, *p* = 2.8e−2), and KIRC (OS: HR = 2.12, 95% CI = 1.56 to 2.9, *p* = 1.2e−6). Notably, the expression of *FAM72B* did not show a significant correlation with OS in breast cancer and THCA, nor with RFS in CSCC and KIRC (Figure 4).

High *FAM72B* expression was correlated with improved prognosis in ovarian cancer (OS: HR = 0.73, 95% CI = 0.55 to 0.96, *p* = 2.4e−2; RFS: HR = 0.66, 95% CI = 0.44 to 0.98, *p* = 3.9e−2), STAD (OS: HR = 0.66, 95% CI = 0.46 to 0.94, *p* = 2.2e−2; RFS: HR = 0.4, 95% CI = 0.16 to 1.03, *p* = 4.9e−2), esophageal squamous cell carcinoma (ESCC) (OS: HR = 0.23, 95% CI = 0.1 to 0.57, *p* = 5.7e−4), and THYM (OS: HR = 0.07, 95% CI = 0.01 to 0.6, *p* = 1.7e−3). However, there was no significant relationship between *FAM72B* expression and RFS in ESCC and THYM. In a unique instance, OS data indicated that high *FAM72B* expression in LUSC was correlated with a favorable prognosis, while RFS showed an inverse association (Figure 5). In summary, *FAM72B* may function as a tumor suppressor gene in patients with THYM and STAD, whereas its overexpression may be more intimately linked to an unfavorable prognosis in patients with KIRP, KIRC, LIHC, SARC, LUAD, ACC, LGG, PRAD, and UVM. Overall, these results validated the significance of *FAM72B* in prognostic assessment for certain cancers, indicating its potential as a prognostic biomarker, with the differential prognostic values of its expression alterations being cancer type-specific.

### 3.3. FAM72B Genetic Mutations in Multiple Human Cancers

**Figure 6:**
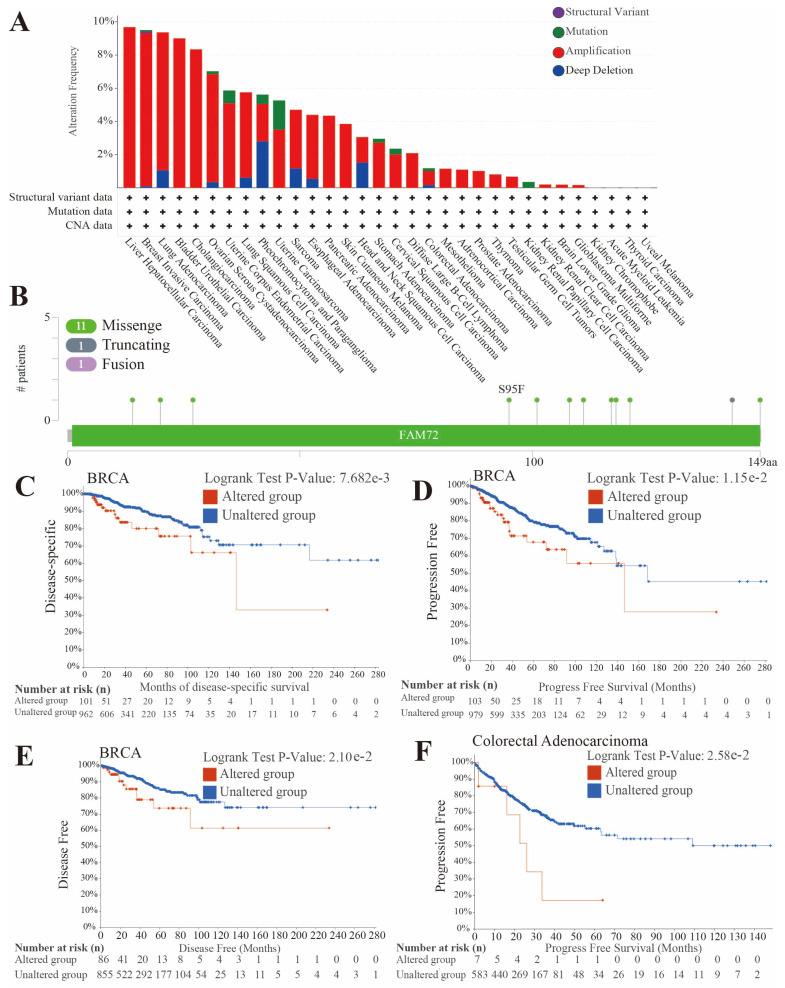
*FAM72B* gene mutations in specific TCGA tumors via the cBioPortal database. (**A**) Types and frequencies of alterations. (**B**) Genetic alterations of *FAM72B*, including types and sites. (**C**–**F**) The correlation between *FAM72B* genetic alterations and cancer prognosis was determined by analyzing survival curves obtained from the cBioPortal database. (**C**–**E**) The correlations of *FAM72B* genetic alterations with poor DSS (**C**), PFS (**D**), and DFS (**E**) in BRCA. (**F**) The correlation between *FAM72B* genetic alterations and poor PFS in colorectal adenocarcinoma. The survival differences between the “altered group” and “unaltered group” were assessed using the Log-rank test; significance threshold: *p* < 0.05. DSS—disease-specific survival; PFS—progression-free survival; DFS—disease-free survival; BRCA—breast invasive carcinoma.

We examined the frequency of genetic alterations in *FAM72B* across various cancers, as well as the specific sites and types of mutations within the *FAM72B* gene using the cBioPortal database (Figure 6A,B). A total of 28 cancer types displayed varying degrees of alterations in the *FAM72B* gene among the 32 tumor types cataloged in the TCGA database. Amplification was the most frequent type of genetic alteration, followed by deep deletion and mutation in 28 cancers. Structural variants were observed exclusively in BRCA. In LIHC, the *FAM72B* gene exhibits the highest frequency of alterations at 9.68%, with amplification being the sole form of alterations. Deep deletion is the major type of genetic alteration in PCPG, with a frequency of 2.81%. UCS presented the highest mutation frequency among all cancers with detectable mutations (Figure 6A). Figure 6B further shows the identification of 13 mutation sites (including 11 missenses, 1 truncation, and 1 fusion), spanning from amino acid positions 0 to 149 within *FAM72B*. Moreover, we assessed the correlation between *FAM72B* genetic alterations and cancer prognosis by analyzing survival curves obtained from the cBioPortal database. The results indicated that patients with BRCA harboring *FAM72B* genetic alterations exhibited worse prognosis in DSS (*p* = 7.682e−3), progression-free survival (PFS) (*p* = 1.15e−2), and disease-free survival (DFS) (*p* = 2.1e−2), while those with *FAM72B* mutations in colorectal adenocarcinoma showed worse PFS (*p* = 2.58e−2) (Figure 6C–F). However, *FAM72B* mutations showed no significant correlation with OS (*p* = 5.06e−2) in BRCA patients, nor with OS (*p* = 2.39e−1) or DSS (*p* = 2.72e−1) in patients with colorectal adenocarcinoma (Figure S2A,B).

### 3.4. Analysis of the Correlation Between Genomic Heterogeneity and Gene Expression of FAM72B in Multiple Cancers

**Figure 7:**
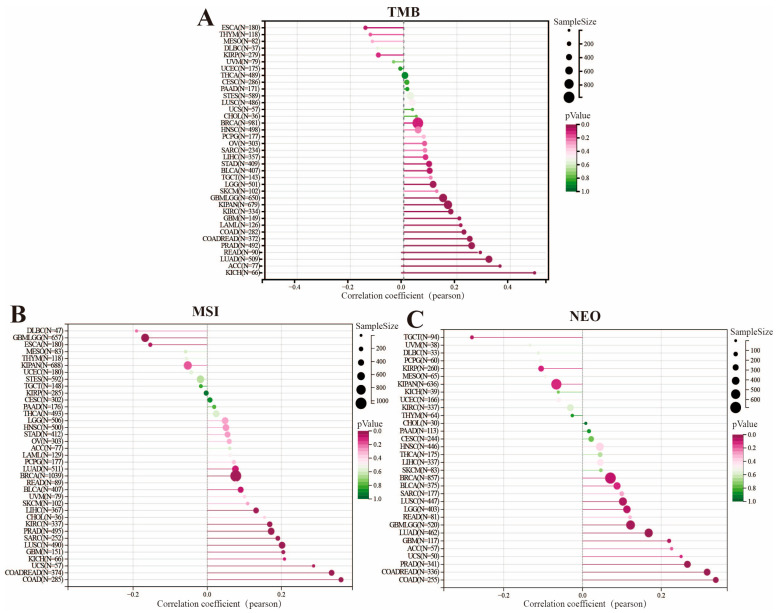
Relationship between tumor mutation burden (TMB) (**A**), microsatellite instability (MSI) (**B**), neoantigen (NEO) (**C**), and *FAM72B* mRNA expression in pan-cancer, visualized by lollipop graphs in SangerBox3.0 online platform.

We investigated the relation between genomic heterogeneity and *FAM72B* gene expression in pan-cancer from the perspectives of TMB and NEO, with the aim of identifying appropriate immunotherapies for the prognosis of cancer patients [53,54,55]. The findings revealed that *FAM72B* expression was positively correlated with TMB levels in all 14 tumor types (LUAD, PRAD, KIPAN, COADREAD, KICH, GBMLGG, COAD, ACC, READ, KIRC, LGG, GBM, LAML, and BLCA), exhibiting significant associations (Figure 7A). As for MSI, *FAM72B* expression showed a significant correlation with MSI in 12 human cancers, with negative correlations observed in GBMLGG and ESCA, and positive correlations in 10 other cancers, including COADREAD, COAD, LUSC, PRAD, KIRC, BRCA, SARC, GBM, LIHC, and UCS (Figure 7B). Additionally, the correlation of *FAM72B* expression with the level of NEO was examined across 32 cancer types. The results indicated that among the eight cancers with significant correlations, *FAM72B* expression was negatively correlated with NEO only in TGCT, while it was positively correlated in the other seven cancers, including COADREAD, COAD, PRAD, LUAD, GBMLGG, LUSC, and GBM (Figure 7C).

### 3.5. Correlation of FAM72B Expression with Immune Checkpoint Genes and Immune Cell Infiltration in Multiple Cancers

**Figure 8:**
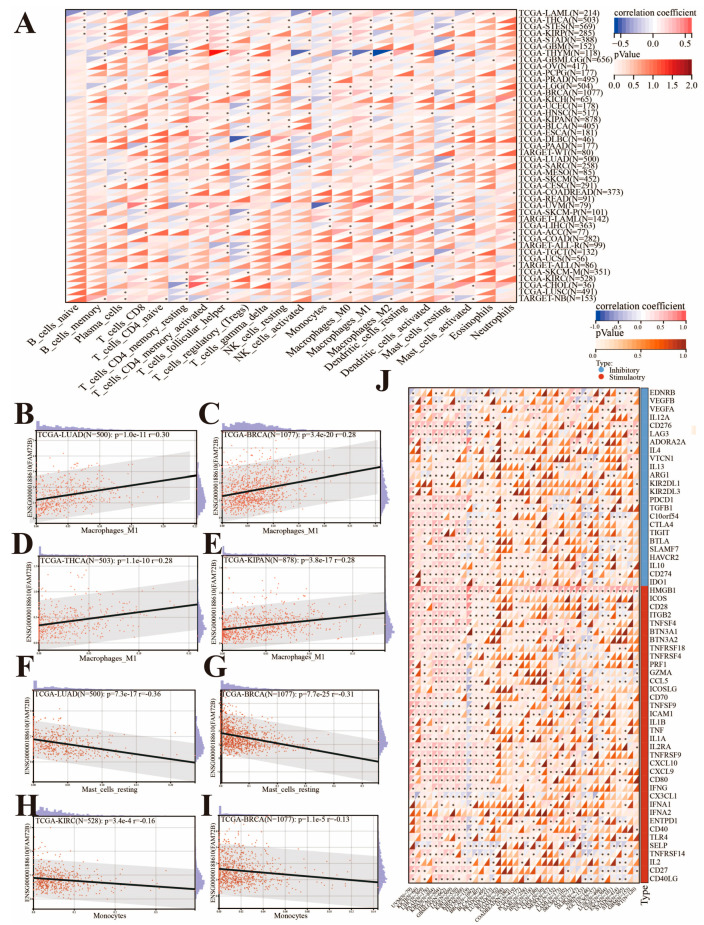
Relationships between immune cell infiltration (**A**) and its partial visualization atlas (**B**–**I**), immune checkpoints (**J**), and *FAM72B* mRNA expression in various tumors on the SangerBox3.0 online platform, where the correlation between *FAM72B* expression and immune cell infiltration was determined by the CIBERSORT algorithm. The upper triangle in each tile indicates coefficients calculated by Pearson’s correlation test, and the lower triangle indicates the log2(x + 1)-transformed *p*-value. (**B**–**E**) Positive correlations of *FAM72B* expression with the infiltration of M1 macrophages in LUAD (**B**), BRCA (**C**), THCA (**D**), and KIPAN (**E**). (**F**–**I**) Negative correlations of *FAM72B* expression with the infiltration of resting mast cells in LUAD (**F**) and BRCA (**G**), and with the infiltration of monocytes in KIRC (**H**) and BRCA (**I**). * indicates a significant *p*-value for correlation, and this designation encompasses the traditional significance levels (* *p* < 0.05). LUAD—lung adenocarcinoma; BRCA—breast invasive carcinoma; THCA—thyroid carcinoma; KIPAN—pan-kidney cohort (KICH + KIRC + KIRP); KIRC—kidney renal clear cell carcinoma.

We analyzed the correlation between *FAM72B* expression and the infiltration of 22 immune cells in multiple tumors using the CIBERSORT algorithm on the SangerBox3.0 online platform (Figure 8A). The findings demonstrated that *FAM72B* expression was significantly linked to the infiltration levels of multiple immune cells in 43 cancer types, excluding UCS. Specifically, *FAM72B* expression levels were negatively correlated with the infiltration levels of macrophages (M0, M1, M2), resting mast cells, activated NK cells, and resting CD4^+^ memory T cells in THYM, while it was positively correlated with follicular helper T cells (Tfh), naive CD4^+^ T cells, resting dendritic cells, and regulatory T cells (Tregs). Additionally, we observed a significant positive correlation between M1 macrophages and *FAM72B* expression in LUAD, BRCA, THCA, and KIPAN (Figure 8B–E). Interestingly, the infiltration of neutrophils was exclusively positively correlated with *FAM72B* expression in KICH, ALL (from the TARGET database), ACC, COAD, COADREAD, and GBMLGG (Figure 8A). We also found that the infiltration levels of resting mast cells were negatively correlated with *FAM72B* expression in LUAD and BRCA. Furthermore, *FAM72B* expression was also negatively correlated with the infiltration levels of monocytes in KICH and BRCA (Figure 8F–I). Then, we further assessed whether there was a correlation between *FAM72B* expression and 60 immune checkpoint genes. The results showed that *FAM72B* expression was predominantly positively associated with most immune checkpoints in UVM, PAAD, MESO, LIHC, KIRP, CHOL, THCA, PCPG, LGG, BLCA, KIRC, OV, GBMLGG, PRAD, KIPAN, and BRCA, and the association was specifically exclusively positive in MESO, CHOL, PCPG, and OV, but predominantly negative in THYM, neuroblastoma (NB, from the TARGET database), and LUSC. In addition, *FAM72B* expression was generally positively related to inhibitory checkpoints like CD276, TGFB1, and VEGFA, and stimulatory checkpoints like HMGB1, TNFSF4, ICAM1, and CXCL10 in most cancers, particularly with HMGB1 showing a positive relation to all of those mentioned except for READ, COADREAD, and COAD (Figure 8J).

### 3.6. Functional Enrichment Analysis of FAM72B-Related Genes

**Figure 9:**
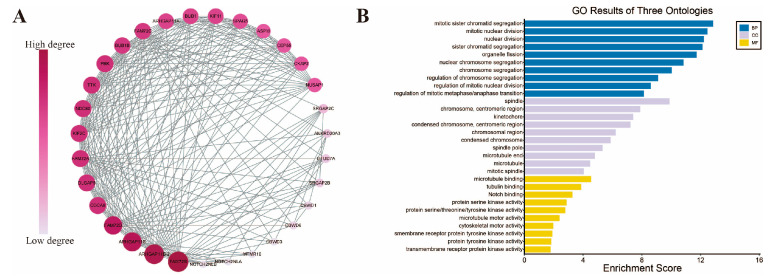
PPI network analysis and GO enrichment analysis of *FAM72B*-related genes. (**A**) The PPI network of *FAM72B* is constructed by the STRING database and Cytoscape software. (**B**) The bar plot of GO enrichment analysis. GO—Gene Ontology; BP—biological process; CC—cellular component; MF—molecular function.

To further investigate the impact of *FAM72B* on the mechanisms of tumor occurrence and progression, we constructed a PPI network comprising 30 *FAM72B*-related genes using the STRING database and subsequently exported the relevant data. The data were then imported into Cytoscape software, where a circular layout was applied for optimal visualization. *FAM72B* was designated as the central node, with other genes arranged around it. Layout parameters, including node spacing and circle radius, were fine-tuned to enhance the clarity of the network. The optimized network was saved, and the positional information of each gene node was documented, thereby providing a solid foundation for subsequent analyses. The results revealed that in the circular layout of Cytoscape, these genes were arranged in a clockwise direction starting from the *FAM72B* node, with their interaction strength with *FAM72B* (measured by the number of connections or interaction scores in the PPI network) progressively decreasing (Figure 9A). Subsequent GO enrichment analyses were performed to elucidate the functional roles of *FAM72B*-related genes in cancer. The results of the GO analysis revealed that *FAM72B*-related genes were significantly enriched in specific biological processes (BPs), with the highest degree of enrichment observed in “mitotic sister chromatid segregation”, “mitotic nuclear division”, “nuclear division”, “sister chromatid segregation”, “organelle fission”, “nuclear chromosome segregation”, and “chromosome segregation”. Furthermore, these genes were predominantly enriched in the cellular component (CC) “spindle”. In the molecular function (MF) analysis, “microtubule binding” was identified as a significant association (Figure 9B). In summary, the genes *TTK*, *DLGAP5*, *BUB1*, *BUB1B*, *SPAG5*, *NUSAP1*, *KIF2C*, *CDCA8*, *KIF11*, and others, as depicted in Figure 9A, are likely associated with “mitotic sister chromatid segregation” and “mitotic nuclear division” in BP, as well as with the “spindle” in CC.

### 3.7. Expression Patterns of FAM72B at the Single-Cell Level

**Figure 10:**
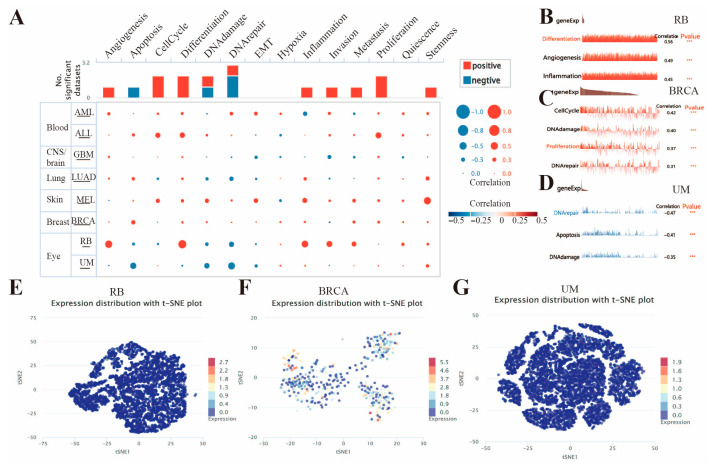
The expression levels of *FAM72B* at the single-cell level in eight types of cancer from the CancerSEA database. (**A**) The functional state of *FAM72B* across eight types of cancer. Red and blue plots indicate positive and negative correlations of *FAM72B* with the functional state, respectively. (**B**–**D**) The relationship between *FAM72B* expression and different functional states in RB (**B**), BRCA (**C**), and UM (**D**) (*** *p* < 0.001). (**E**–**G**) A T-SNE diagram visualizing *FAM72B* expression in single cells from RB (**E**), BRCA (**F**), and UM (**G**). RB—retinoblastoma; BRCA—breast invasive carcinoma; UM—uveal melanoma.

To clarify the expression correlations of the *FAM72B* gene in various cancer types, we explored its functional states using the CancerSEA database. *FAM72B* was studied at the single-cell level in eight types of cancer, including retinoblastoma (RB), BRCA, uveal melanoma (UM), ALL, melanoma (MEL), acute myeloid leukemia (AML), GBM, and LUAD, with significant correlations observed only in RB, BRCA, UM, ALL, and MEL. In RB, *FAM72B* expression correlated positively with angiogenesis, differentiation, inflammation, invasion, and metastasis, but negatively with DNA repair. Its correlation with DNA damage, DNA repair, and proliferation was substantially positive, but negative with the cell cycle in BRCA. In addition, it was negatively correlated with apoptosis, DNA damage, and DNA repair in UM. However, it showed no significant correlation with 3 of the 14 functional states listed for the cancers, namely the epithelial–mesenchymal transition (EMT), hypoxia, and quiescence (Figure 10A). Figure 10B–D further visually demonstrate the significant correlation between *FAM72B* expression and angiogenesis, differentiation, and inflammation in RB, DNA damage, DNA repair, and proliferation in BRCA, as well as apoptosis, DNA damage, and DNA repair in UM. Furthermore, we employed the T-SNE diagram to visualize the expression of *FAM72B* at the single-cell level in RB, BRCA, and UM (Figure 10E–G).

## 4. Discussion

Existing studies have indicated that *FAM72B* is overexpressed in various tumor types, including OSCC, clear cell renal cell carcinoma (ccRCC), LUAD, and glioblastoma multiform (GBM). In OSCC and ccRCC, high expression of *FAM72B* is associated with poor prognosis and is an independent prognostic factor [22,56]. In LUAD, the expression level of *FAM72B* is closely related to the infiltration of immune cells in the tumor microenvironment [29]. In GBM, somatic mutation data analysis of *FAM72B* has identified a new set of GBM-specific genes that are potential therapeutic targets [12].

Although *FAM72B* plays a significant role in cancer, to the best of our knowledge, there have been no studies published to date regarding its pan-cancer analysis in humans. In this study, we employed a series of bioinformatics methods to systematically analyze the correlation between *FAM72B* expression and various aspects, including multiple human tumor types, clinical pathological parameters, prognostic value, gene mutations, genomic heterogeneity, immune checkpoint genes, immune cell infiltration levels, and single-cell levels, in order to explore the potential oncogenic or tumor-suppressive roles of *FAM72B*. We found that, compared with normal adjacent tissues, *FAM72B* is overexpressed in the majority of cancers, including PRAD and GBM, which is consistent with previous research findings [12,31,57]; however, its expression is downregulated in THCA and KICH. Given that the Wnt/β-catenin signaling pathway plays a crucial role in biological processes such as cell proliferation, differentiation, and migration, its abnormal activation in KICH may not only promote malignant behaviors of cancer cells, such as proliferation and invasion, but may also indirectly lead to the downregulation of *FAM72B* expression by disrupting the normal mechanisms of gene expression regulation [58]. In multiple tumors, the expression of *FAM72B* increases with the progression of tumor staging, with the most significant increases observed in KIPAN, KIRC, ACC, and PRAD, which typically indicates that this gene is associated with tumor progression and may portend a poorer prognosis. However, in the stage classification, the expression level of *FAM72B* gradually decreases with the progression of OV staging, which is in contrast to the increasing trend observed in the grade classification. This indicates that *FAM72B* in OV functions as a complex prognostic marker with a dual role, with corresponding treatment strategies likely being more effective in the early or intermediate stages. In advanced OV, a combination of multiple therapeutic approaches may be necessary to enhance treatment efficacy, and further research is required to clarify the specific mechanisms. In THCA, *FAM72B* expression is higher in normal tissues but downregulated in tumor tissues, with higher expression in low-invasive tumors and the highest expression in the N1 stage (lymph node metastasis). This suggests that *FAM72B* likely plays a significant role in the normal physiological functions of thyroid tissue. In the early stages of tumors, it may exert protective effects by maintaining cell differentiation, inhibiting invasion and metastasis, and modulating signaling pathways (e.g., PI3K/AKT/mTOR) [59], thereby restricting tumor malignancy progression. However, in the N1 stage, the high expression of *FAM72B* may be an adaptive change to the lymph node microenvironment, facilitating tumor cell survival and proliferation, potentially associated with the reactivation of signaling pathways such as Ras/Raf/MEK/ERK [60]. Therefore, *FAM72B* may function as a tumor suppressor gene in the early stages. Its expression changes are closely related to tumor metastasis and can serve as a potential biomarker for assessing metastatic risk and prognosis. Next, we further analyzed the relationship between *FAM72B* mRNA expression levels and tumor progression, and comprehensively evaluated the correlation between *FAM72B* expression and pan-cancer prognostic indicators such as OS, RFS, DSS, DFI, and PFI using the pan-cancer platform SangerBox3.0, the GEPIA2.0 database, and the Kaplan–Meier plotter database. OS is the most direct and comprehensive indicator for assessing the effectiveness of cancer treatments, directly reflecting the patient’s survival time and serving as the most commonly used primary endpoint in clinical trials [61,62]. RFS is an important indicator for measuring treatment effectiveness, and is particularly crucial in the assessment of early-stage cancer treatment outcomes [63]. DSS excludes the impact of non-disease-related deaths, providing a more accurate assessment of the specific disease’s effect on patient survival, and is particularly useful in research on specific cancer types [64]. DFI is primarily used to assess the patient’s disease-free state, especially in evaluating the long-term effects of curative treatments [65]. Compared to RFS, PFI is more important in the assessment of treatment effectiveness in advanced or metastatic cancers, as it helps us to understand the effect of the treatment in delaying cancer progression [66]. In our study, we found that overexpression of *FAM72B* was associated with adverse prognosis in KIRP, KIRC, LIHC, SARC, LUAD, ACC, LGG, PRAD, and UVM. Taking LIHC and UVM as examples, *FAM72B* may promote tumor cell proliferation in LIHC by activating cell cycle-related genes (e.g., Cyclin D1) and accelerating the cell cycle process [67]. *FAM72B* may also participate in the activation of signaling pathways that facilitate tumor progression, such as the PI3K/AKT/mTOR pathway [68]. The high expression of *FAM72B* in LIHC may be associated with the characteristics of cancer stem cells [69]. *FAM72B* may contribute to the formation and maintenance of cancer stem cells, thereby increasing the risk of tumor recurrence and metastasis and leading to poor prognosis. In UVM, *FAM72B* may reshape the extracellular matrix, thereby increasing the stiffness and density of the tumor microenvironment, which in turn inhibits the infiltration of monocytes and maintains the immunosuppressive characteristics of the tumor microenvironment [70,71]. However, in THYM and STAD, it served as a protective factor. In THYM, the high expression of *FAM72B* may enhance the anti-tumor capacity of the immune system by promoting the infiltration of Tfh cells, thereby improving patient prognosis [72,73]. Interestingly, Kaplan–Meier curve analysis suggested that the high expression of *FAM72B* in LUSC was associated with a favorable prognosis of OS, while RFS showed an inverse association. This result indicates that such a high expression may play a positive role in certain biological processes, such as cell survival and proliferation, thereby extending OS. However, it may also promote tumor invasiveness and recurrence, leading to a decrease in RFS. This discrepancy suggests that evaluating *FAM72B* as a prognostic biomarker requires considering multiple survival indicators and further investigating its specific mechanisms of action in tumors to better understand its value and potential clinical applications in LUSC prognosis.

The data on the frequency of *FAM72B* genetic alterations indicate that amplification is the most common type of genetic alteration, with the highest frequency observed in LIHC at 9.68%. Structural variants were exclusively observed in BRCA. The types of specific mutation sites within the *FAM72B* gene are predominantly missense mutations. We further evaluated the correlation between *FAM72B* gene mutations and cancer prognosis. The results showed that patients with BRCA and colorectal adenocarcinoma harboring *FAM72B* genetic alterations had a poorer prognosis.

TMB has been approved by the FDA as a biomarker for assessing the therapeutic effects of immune checkpoint inhibitors (ICIs). Compared with cancer patients who have not received ICI treatment, those with high TMB who have received ICI treatment exhibit significantly improved OS and PFS, indicating a better prognosis [74,75,76]. Currently, solid tumors that exhibit both microsatellite instability–high (MSI-H) and mismatch repair deficiency (dMMR) typically demonstrate high levels of lymphocytic infiltration, which renders them highly responsive to ICIs, thereby achieving favorable immunotherapy outcomes [77,78,79]. It is worth noting that neoantigens, which are tumor-specific proteins, form the basis for the personalized vaccines that can achieve individualized and precise immunotherapy, thereby offering a new direction for cancer treatment [80,81,82]. Therefore, in our study, we revealed that the expression levels of *FAM72B* are positively correlated with the levels of TMB, MSI, and NEO in most cancers, suggesting a possible higher response rate to ICIs among patients with high *FAM72B* expression. This finding may provide a new biomarker for identifying potential beneficiaries of ICI therapy, contributing to improved treatment outcomes and patient prognosis. An increasing number of studies have demonstrated that the TIME plays a crucial role in tumor malignancy progression, immune escape, and treatment resistance. Targeting immune checkpoints and immune-infiltrating cells within the TIME can enhance anti-tumor immune responses and improve the efficacy of ICIs, with combination therapies such as PD-1/CTLA-4 dual blockade emerging as a principal strategy to enhance the therapeutic outcomes of ICI [83,84,85]. This study found that *FAM72B* expression is generally positively correlated with the expression of most immune checkpoint genes across pan-cancers, particularly for HMGB1, which showed a positive relation to all except for READ, COADREAD, and COAD. Although HMGB1 is not a conventional immune checkpoint molecule, and its inhibitors are predominantly used in the treatment of autoimmune diseases [86], it plays significant role in immune modulation and tumor immunotherapy. Specifically, HMGB1 is implicated in inhibiting the proliferation of CRC cells [87], participating in autophagy [88,89], and contributing to cancer progression through the induction of pro-inflammatory factors [90,91]. This may suggest that the regulatory mechanisms or functions of FAM72B and HMGB1 differ in CRC, or that their roles in LUAD may be influenced by other factors. In LUAD, FAM72B and HMGB1 may jointly activate the PI3K/AKT signaling pathway, thereby promoting the proliferation and survival of tumor cells. The overexpression of FAM72B may upregulate HMGB1 expression or enhance its functionality, indirectly influencing fibroblasts within the tumor microenvironment and facilitating the activation, migration, and proliferation of cancer-associated fibroblasts (CAFs) [29,68,92]. Subsequently, we investigated the association of *FAM72B* mRNA expression with the infiltration levels of various immune cells in pan-cancer. The results show that *FAM72B* correlates positively with Tfh cells in 21 tumor types (e.g., THYM, KIRP, UVM) and M1 macrophages in 19 tumor types (e.g., LUAD, BRCA, THCA, and KIPAN), but negatively with resting mast cells in 23 tumor types (e.g., THYM, BRCA, LUAD) and monocytes in 20 tumor types (e.g., UVM, KICH, BRCA, KIRC). Previous studies have demonstrated that tumor-infiltrating mast cells are associated with immune evasion in gastric cancer (GC) [93] and melanoma [94], leading to resistance to anti-PD-1 therapy, and are correlated with prognosis in HCC [95], where a high infiltration of resting mast cells is linked to poorer survival outcomes and adverse prognosis [96]. Recent studies have demonstrated that late-stage M1-like macrophages activated by immunotherapy-activated T cells are positively correlated with the response to ICI therapy, which is critical for effective tumor control [97]. Moreover, one study has revealed that MANF supplementation in monocyte-derived macrophages (MDMs) enhances the interaction between heat shock factor 1 (HSF1) and heat shock protein 70 (HSP-70), thereby inhibiting the nuclear import of HSF1, which in turn negatively regulates the expression of HSP70-1 in macrophages, reprograms tumor-associated macrophages (TAMs) into an M1-like phenotype, and ultimately suppresses HCC neovascularization to achieve therapeutic efficacy [98]. In 2022, the Cao Xuetao team first revealed that M2-like TAMs are polarized via the glucose utilization mechanism in TAMs, thereby exerting a pro-tumor metastasis effect [99].

The results of the GO analysis indicated that *FAM72B*-related genes exhibited the highest degree of enrichment in the biological process (BP), with predominant enrichment in “mitotic sister chromatid segregation”, “mitotic nuclear division”, “nuclear division”, “sister chromatid segregation”, “organelle fission”, “nuclear chromosome segregation”, and “chromosome segregation”. These findings suggest that *FAM72B* may play a crucial role in mitotic processes, which are essential for cell division and tumor progression [12,23,34]. Moreover, the primary enrichment of these genes in the cellular component “spindle” indicates potential involvement in the structural integrity and function of the mitotic spindle. In the analysis of molecular function (MF), the association of these genes with “microtubule binding” was noted, suggesting that *FAM72B*-related genes may interact with microtubules to facilitate proper mitotic spindle formation and function [100,101,102]. These findings offer novel insights into the specific mechanisms by which *FAM72B*-related genes contribute to tumorigenesis and tumor progression. Specifically, *NUSAP1* activates the expression of *lactate dehydrogenase A* (*LDHA*) by binding to c-Myc and hypoxia-inducible factor (HIF-1α), thereby promoting glycolysis and lactate production, and lactate in turn upregulates the expression of NUSAP1 protein by lysine lactylated modification, forming a NUSAP1–LDHA–glycolysis–lactate feedforward loop that enhances the Warburg effect and metastatic potential in pancreatic ductal adenocarcinoma (PDAC) [103]. Recent studies have, for the first time, identified *SPAG5* as a direct transcriptional target of the YAP-TAZ-TEAD axis and a direct target of *miR-10b-3p*, elucidating the mechanism by which the YAP-TAZ-TEAD axis in the Hippo signaling pathway upregulates *SPAG5* expression by inhibiting *miR-10b-3p*, thereby promoting the proliferation and migration of breast cancer cells, and thus providing a novel therapeutic target for the treatment of breast cancer [104]. Recent studies have elucidated two pivotal mechanisms of *DLGAP5* in bladder cancer. On one hand, DLGAP5 directly interacts with E2F1 and stabilizes it by inhibiting its ubiquitination through the deubiquitinating enzyme USP11, thereby forming a DLGAP5-USP11-E2F1 feedback loop that promotes the proliferation and migration of bladder cancer cells and drives the progression of bladder cancer [105]. On the other hand, DLGAP5 interacts with the deubiquitinating enzyme USP11 to stabilize MYC protein, which in turn upregulates the expression of glycolysis-related genes in bladder cancer cells, forming a DLGAP5-USP11-MYC-positive feedback loop that enhances chemoresistance to gemcitabine in bladder cancer cells [106].

Recently, Bai Fan from Peking University and fellow researchers constructed the largest-scale pan-cancer brain metastasis single-cell atlas to date, elucidating the characteristics of brain metastases (BrMs) and the surrounding microenvironment [107], which further illustrates that the advancement of single-cell sequencing technology [108] has significantly propelled the depth and precision of pan-cancer research. In our study, single-cell sequencing results indicated significant associations between *FAM72B* and the biological functional states of various cancers. Considering UM as an example, the expression of *FAM72B* in UM is significantly negatively correlated with DNA repair, apoptosis, and DNA damage, implying that high *FAM72B* expression may drive tumor progression and suppress apoptosis, thereby indicating a poorer prognosis. Accordingly, *FAM72B* could serve as a potential prognostic biomarker and therapeutic target for UM patients. It may be beneficial to inhibit tumor initiation and progression by restoring the DNA repair capacity and apoptosis mechanisms of tumor cells through modulating *FAM72B* expression via gene-editing technologies or developing drugs targeting its associated pathways.

## 5. Conclusions

In summary, using multiple authoritative bioinformatics technologies, we comprehensively investigated *FAM72B*’s expression patterns, prognostic significance, and immunomodulatory functions in pan-cancer. Our findings indicate that *FAM72B* is likely to serve as a potential prognostic biomarker and therapeutic target for diverse cancers. Although its potential role in cancer was revealed, the specific molecular mechanisms remain to be further explored. This study offers novel insights into the functions of *FAM72B* in tumor development and treatment, laying the foundation for the future development of personalized treatment plans.

## Figures and Tables

**Table 1:** Abbreviations and full names of 34 types of cancers in the TCGA and TARGET databases.

Abbreviation	Full Name
ACC	Adrenocortical carcinoma
ALL	Acute lymphoblastic leukemia
BLCA	Bladder urothelial carcinoma
BRCA	Breast invasive carcinoma
CESC	Cervical squamous cell carcinoma and endocervical adenocarcinoma
CHOL	Cholangiocarcinoma
COAD	Colon adenocarcinoma
COADREAD	Colon adenocarcinoma/rectum adenocarcinoma
ESCA	Esophageal carcinoma
GBM	Glioblastoma multiforme
GBMLGG	Low-grade glioma and glioblastoma
HNSC	Head and neck squamous cell carcinoma
KICH	Kidney chromophobe
KIRC	Kidney renal clear cell carcinoma
KIPAN	Pan-kidney cohort (KICH + KIRC + KIRP)
KIRP	Kidney renal papillary cell carcinoma
LAML	Acute myeloid leukemia
LGG	Brain lower-grade glioma
LIHC	Liver hepatocellular carcinoma
LUAD	Lung adenocarcinoma
LUSC	Lung squamous cell carcinoma
OV	Ovarian serous cystadenocarcinoma
PAAD	Pancreatic adenocarcinoma
PCPG	Pheochromocytoma and paraganglioma
PRAD	Prostate adenocarcinoma
READ	Rectum adenocarcinoma
SKCM	Skin cutaneous melanoma
STAD	Stomach adenocarcinoma
STES	Stomach and esophageal carcinoma
TGCT	Testicular germ cell tumors
THCA	Thyroid carcinoma
UCEC	Uterine corpus endometrial carcinoma
UCS	Uterine carcinosarcoma
WT	High-risk Wilms tumor

**Table 2:** Correlation of *FAM72B* mRNA expression and prognosis in various cancers by Kaplan–Meier plotter.

Cancer Type	Overall Survival		Recurrence-Free Survival	
	Hazard Ratio	*p*-Value	Hazard Ratio	*p*-Value
KIRP	5.46 (2.99–9.98)	6.4e−10	5.74 (2.59–12.69)	1.2e−6
LIHC	1.9 (1.34–2.68)	2.2e−4	1.69 (1.19–2.4)	2.7e−3
LUAD	2.11 (1.54–2.89)	2e−6	1.66 (1.03–2.68)	3.4e−2
UCEC	2.03 (1.34–3.08)	6.8e−4	2.44 (1.37–4.35)	1.8e−3
SARC	2 (1.29–3.09)	1.5e−3	1.91 (1.07–3.42)	2.6e−2
CSCC	1.7 (1.05–2.76)	2.8e−2	1.65 (0.75–3.64)	2.1e−1
KIRC	2.12 (1.56–2.9)	1.2e−6	2.4 (0.76–7.57)	1.2e−1
THCA	2.09 (0.78–5.63)	1.3e−1	2.52 (1.16–5.49)	1.6e−2
Breast Cancer	1.32 (0.95–1.84)	9.8e−2	1.96 (1.06–3.62)	2.8e−2
Ovarian Cancer	0.73 (0.55–0.96)	2.4e−2	0.66 (0.44–0.98)	3.9e−2
STAD	0.66 (0.46–0.94)	2.2e−2	0.4 (0.16–1.03)	4.9e−2
LUSC	0.73 (0.56–0.96)	2.2e−2	1.68 (1–2.83)	4.9e−2
ESCC	0.23 (0.1–0.57)	5.7e−4	0.43 (0.17–1.14)	8.1e−2
THYM	0.07 (0.01–0.6)	1.7e−3	NA	NA

NA—not available; *p* < 0.05 is considered statistically significant.

## Data Availability

The data utilized in the manuscript are publicly accessible via the following links: TCGA (https://portal.gdc.cancer.gov/), GTEx (https://www.gtexportal.org/home/index.html), SangerBox3.0 platform (http://sangerbox.com/), HPA database (https://www.proteinatlas.org/), Kaplan–Meier Plotter (https://kmplot.com/analysis/), GEPIA2.0 (http://gepia2.cancer-pku.cn/#index), cBioPortal database (https://www.cbioportal.org/), STRING database (https://cn.string-db.org/), Cytoscape (https://cytoscape.org/), and CancerSEA database (http://biocc.hrbmu.edu.cn/CancerSEA/). All accessed on 12 September 2025.

## Supplementary Materials

The following supporting information can be downloaded at https://www.mdpi.com/article/10.3390/genes16101140/s1, Figure S1. Kaplan-Meier survival curve analysis of the prognostic significance of high and low expression of *FAM72B* in 14 types of human cancers using the GEPIA2.0 database. (A–J) Correlation of high *FAM72B* expression with poor OS and DFS in ACC (A,B), KIRP (C,D), LGG (E,F), LIHC (G,H), and SARC (I,J). (K–M) Correlation of high *FAM72B* expression with poor OS in KIRC (K), LUAD (L), and MESO (M). (N–Q) Correlation of high *FAM72B* expression with poor DFS in PAAD (N), PRAD (O), KICH (P), and UVM (Q). (R,S) Correlation of high FAM72B expression with better OS in LUSC (R) and THYM (S). The median value selected as group cutoff to split high-expression and low-expression cohorts of FAM72B; significance threshold of *p* < 0.05 to determine statistical significance. Assessment of the significance of expression differences in FAM72B using HR. OS, overall survival; DFS, disease-free survival; ACC, adrenocortical carcinoma; KIRP, kidney renal papillary cell carcinoma; LGG, brain lower grade glioma; LIHC, liver hepatocellular carcinoma; SARC, sarcoma; KIRC, kidney renal clear cell carcinoma; LUAD, lung adenocarcinoma; MESO, mesothelioma; PAAD, pancreatic adenocarcinoma; PRAD, Prostate adenocarcinoma; KICH, kidney chromophobe; UVM, uveal melanoma; LUSC, lung squamous cell carcinoma; THYM, thymoma; HR, hazard ratio. Figure S2. Kaplan-Meier survival curve analysis of the prognostic significance of *FAM72B* genetic alterations in breast invasive carcinoma (BRCA) and colorectal adenocarcinoma using the cBioPortal database. (A) No significant correlation between *FAM72B* genetic alterations and OS in BRCA. (B,C) No significant correlation of *FAM72B* genetic alterations with OS (B) or DSS (C) in colorectal adenocarcinoma. The survival differences between “altered group” and “unaltered group” assessed by the Log-rank test; significance threshold: *p* < 0.05. OS, overall survival; DSS, disease-specific survival.
