# Supplementary material for: Spotlight on FAM72B: Pan-Cancer Expression Profiles and Its Potential as a Prognostic and Immunotherapeutic Biomarker
Source: Genes (Basel). 2025 Sep 26;16(10):1140. doi: 10.3390/genes16101140 (PMC12564144; doi:10.3390/genes16101140)
Supplement: Supplementary file 1 [file genes-16-01140-s001.zip › genes-3842009-supplementary.pdf]

Supplementary Materials

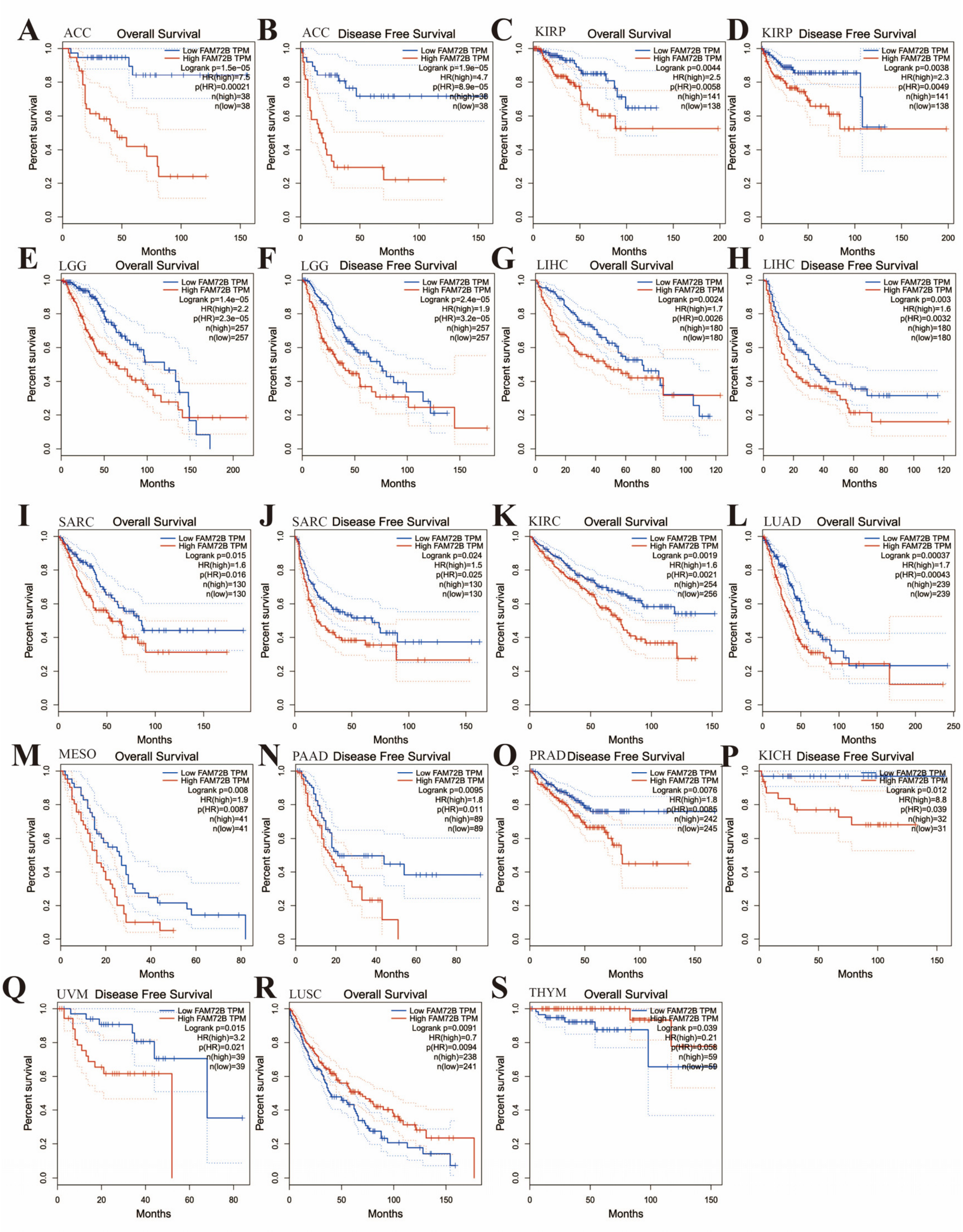

Figure S1. Kaplan-Meier survival curve analysis of the prognostic significance of high and low expression of *FAM72B* in 14 types of human cancers using the GEPIA2.0 database. (A-J) Correlation of high *FAM72B* expression with poor OS and DFS in ACC (A, B), KIRP (C, D), LGG (E, F), LIHC (G, H), and SARC (I. J). (K-M) Correlation of high *FAM72B* expression

with poor OS in KIRC (K), LUAD (L), and MESO (M). (N-Q) Correlation of high *FAM72B* expression with poor DFS in PAAD (N), PRAD (O), KICH (P), and UVM (Q). (R, S) Correlation of high *FAM72B* expression with better OS in LUSC (R) and THYM (S). The median value selected as group cutoff to split high-expression and low-expression cohorts of *FAM72B*; significance threshold of P < 0.05 to determine statistical significance. Assessment of the significance of expression differences in FAM72B using HR. OS, overall survival; DFS, disease-free survival; ACC, adrenocortical carcinoma; KIRP, kidney renal papillary cell carcinoma; LGG, brain lower grade glioma; LIHC, liver hepatocellular carcinoma; SARC, sarcoma; KIRC, kidney renal clear cell carcinoma; LUAD, lung adenocarcinoma; MESO, mesothelioma; PAAD, pancreatic adenocarcinoma; PRAD, Prostate adenocarcinoma; KICH, kidney chromophobe; UVM, uveal melanoma; LUSC, lung squamous cell carcinoma; THYM, thymoma; HR, hazard ratio.

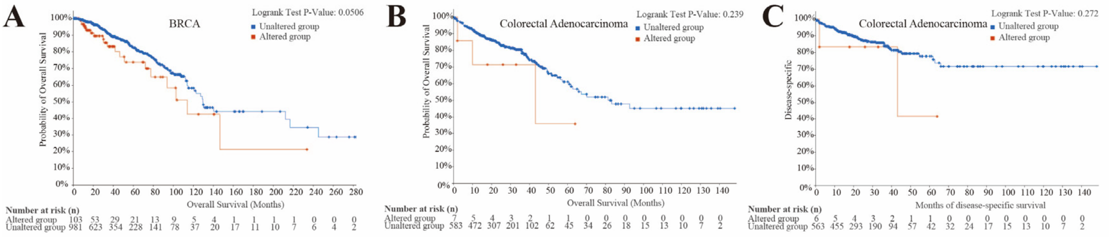

Figure S2. Kaplan-Meier survival curve analysis of the prognostic significance of *FAM72B* genetic alterations in breast invasive carcinoma (BRCA) and colorectal adenocarcinoma using the cBioPortal database. (A) No significant correlation between *FAM72B* genetic alterations and OS in BRCA. (B, C) No significant correlation of *FAM72B* genetic alterations with OS (B) or DSS (C) in colorectal adenocarcinoma. The survival differences between "altered group" and “unaltered group” assessed by the Log-rank test; significance threshold: P < 0.05. OS, overall survival; DSS, disease-specific survival.
